# Post-Concussion Syndrome and Chronic Traumatic Encephalopathy: Narrative Review on the Neuropathology, Neuroimaging and Fluid Biomarkers

**DOI:** 10.3390/diagnostics12030740

**Published:** 2022-03-18

**Authors:** Ioannis Mavroudis, Dimitrios Kazis, Rumana Chowdhury, Foivos Petridis, Vasiliki Costa, Ioana-Miruna Balmus, Alin Ciobica, Alina-Costina Luca, Iulian Radu, Romeo Petru Dobrin, Stavros Baloyannis

**Affiliations:** 1 Department of Neuroscience, Leeds Teaching Hospitals, NHS Trust, Leeds LS2 9JT, UK; i.mavroudis@nhs.net (I.M.); rumana.chowdhury@nhs.net (R.C.); 2 Laboratory of Neuropathology and Electron Microscopy, Aristotle University of Thessaloniki, 54634 Thessaloniki, Greece; v.cossta@auth.gr (V.C.); sibh844@otenet.gr (S.B.); 3 Research Institute for Alzheimer’s Disease and Neurodegenerative Diseases, Heraklion Langada, 57200 Thessaloniki, Greece; 4 Third Department of Neurology, Aristotle University of Thessaloniki, 57010 Thessaloniki, Greece; dimitrios.kazis@gmail.com (D.K.); f_petridis83@yahoo.gr (F.P.); 5 Department of Exact Sciences and Natural Sciences, Institute of Interdisciplinary Research, “Alexandru Ioan Cuza” University of Iași, 700057 Iași, Romania; balmus.ioanamiruna@yahoo.com; 6 Department of Biology, Faculty of Biology, Alexandru Ioan Cuza University, 700506 Iași, Romania; 7 Faculty of Medicine, “Grigore T. Popa” University of Medicine and Pharmacy, 700115 Iași, Romania; raduiuli@gmail.com

**Keywords:** post-concussion syndrome, chronic traumatic encephalopathy, neuropathology, neuroimaging, fluid biomarkers

## Abstract

Traumatic brain injury is a significant public health issue and represents the main contributor to death and disability globally among all trauma-related injuries. Martial arts practitioners, military veterans, athletes, victims of physical abuse, and epileptic patients could be affected by the consequences of repetitive mild head injuries (RMHI) that do not resume only to short-termed traumatic brain injuries (TBI) effects but also to more complex and time-extended outcomes, such as post-concussive syndrome (PCS) and chronic traumatic encephalopathy (CTE). These effects in later life are not yet well understood; however, recent studies suggested that even mild head injuries can lead to an elevated risk of later-life cognitive impairment and neurodegenerative disease. While most of the PCS hallmarks consist in immediate consequences and only in some conditions in long-termed processes undergoing neurodegeneration and impaired brain functions, the neuropathological hallmark of CTE is the deposition of p-tau immunoreactive pre-tangles and thread-like neurites at the depths of cerebral sulci and neurofibrillary tangles in the superficial layers I and II which are also one of the main hallmarks of neurodegeneration. Despite different CTE diagnostic criteria in clinical and research approaches, their specificity and sensitivity remain unclear and CTE could only be diagnosed post-mortem. In CTE, case risk factors include RMHI exposure due to profession (athletes, military personnel), history of trauma (abuse), or pathologies (epilepsy). Numerous studies aimed to identify imaging and fluid biomarkers that could assist diagnosis and probably lead to early intervention, despite their heterogeneous outcomes. Still, the true challenge remains the prediction of neurodegeneration risk following TBI, thus in PCS and CTE. Further studies in high-risk populations are required to establish specific, preferably non-invasive diagnostic biomarkers for CTE, considering the aim of preventive medicine.

## 1. Introduction

Traumatic brain injury (TBI) is a significant global public health issue and represents the main contributor to death and disability among all trauma-related injuries [1]. Sixty-nine million patients are estimated to suffer a traumatic brain injury each year worldwide [2]. In the United States of America, 1.5 million traumatic brain injuries occur every year, 75% of which are mild [3]. 

Non-recurring concussions and mild brain injuries due to TBI usually do not impose chronic consequences on the brain tissues of the patients [4]. The effects of such traumatism are usually short-termed, and the symptoms alleviate over some weeks or months. In this way, the post-concussive syndrome (PCS) following a mild traumatic brain injury could exhibit mild symptoms that last no more than four weeks [5]. However, in some cases, persistent PCS symptomatology could predict subsequent brain damage or risk for further comorbid conditions [6,7]. Thus, repetitive brain injuries are linked to increased risk of later-life cognitive impairment and neurodegenerative disorders, including Chronic Traumatic Encephalopathy [8].

Repetitive mild head injuries (RMHI) can be observed in athletes, military veterans, martial arts practitioners, victims of physical abuse, and epileptic patients. The effects of traumatic brain injury (TBI) or RMHI in later life are not well understood. However, recent studies suggested that even mild head injuries could increase the risk of later-life cognitive impairment and neurodegenerative disease [9]. Even less severe traumatic brain injuries have been linked with an increased risk of dementia and reduced age of onset for Alzheimer’s disease (AD) [9]. Furthermore, it was demonstrated that repeated TBIs could increase the risk for neurodegenerative processes, such as the development of Chronic Traumatic Encephalopathy (CTE) [10,11,12,13,14,15,16,17,18,19,20,21]. In this way, it was shown that the neuropathological hallmark of CTE is the deposition of p-tau immunoreactive pre-tangles and thread-like neurites at the depths of cerebral sulci, and neurofibrillary tangles in the superficial layers I and II [22], which are also some of the most important features of initial neurodegeneration processes [23,24]. However, CTE could only be diagnosed post-mortem, and although different diagnostic criteria can be used for clinical and research purposes, their specificity and sensitivity are unclear [25,26,27]. 

A possible link between TBI/RMHI and CTE or early dementia has widespread implications for predisposed individuals in which TBI/RMHI are prone to occur more often or repeatedly. Neurodegeneration was not the only risk associated with the RMHI occurrence, as some recent studies showed that depression, anxiety, post-traumatic stress disorder, sleep disorders, as well as cardiovascular disorders, metabolic syndromes, chronic pain, musculoskeletal fragility, and other heterotypic disorders were also associated with the subsequent long-term consequences of traumatic brain injuries [28,29,30,31,32]. 

In this context, the current work aims to describe the possible correlations between the mentioned forms of traumatic brain injury consequences and the risk for the early onset of neurodegenerative processes. In the context of preventive medicine, the emphasis falls on the early detection of any clues that could indicate the initiation of a pathological process, the diagnostic biomarkers in traumatic brain injuries. We aim to discuss this matter as considering their relevance in predicting the possible occurrence of neurodegenerative processes in CTE and the risk associated with repetitive traumatic brain injuries for exposed individuals.

## 2. Methodology

Information was gathered and selected by conducting primary scientific research databases (e.g., ScienceDirect, PubMed/Medline, Embase, and Google Scholar) screening for studies regarding the traumatic brain injury effects, post-concussion syndrome, and chronic traumatic encephalopathy (between 1975 and 2021). We selected only the articles available in the English language and screened them by title, keywords, abstract, and full content. Keywords such as “brain injury”, “repetitive brain injury”, “traumatic brain injury”, “post concussive syndrome”, “chronic traumatic encephalopathy”, “neuroimaging”, “fluid biomarkers”, “mechanisms”, “neuropathology”, and combinations of those were used to screen the databases for relevant studies. The selection process was conducted by multiple separate researchers, and the common consent of the authors settled all differences in opinion.

## 3. Clinical Diagnosis and Definition

### 3.1. Concussion, TBI, and RMHI

Traumatic brain injuries can cause loss of consciousness, post-traumatic amnesia, disorientation and confusion, and new-onset neurological symptoms such as post-traumatic epilepsy, anosmia, or hemiparesis. These symptoms present immediately after the occurrence of the TBI, or immediately after the recovery of consciousness and may persist past the acute post-injury period [33]. The clinical entity defined by the persistent neurological symptoms following a TBI is post-concussion syndrome (PCS).

The pathophysiology of concussion is not clearly understood. It is believed that stretching and disruption of neuronal and axonal cell membranes occur after a head injury, leading to neurometabolic cascade activation preceding neuronal and axonal injury and death and potentially to neuroinflammation and microglia activation [34,35]. Considering these aspects, many classifications systems were proposed. However, only a few are still widely used, possibly due to the fact that most of the classification and diagnostic criteria for concussion consequences and TBI are instead based on clinical observations and symptomatology. Cantu et al. 2006 thoroughly described most of these classification systems and provided evidence on their grounds and use direction [36]. 

There are different classification systems for TBIs, based on severity, pathoanatomic type, outcome, and prognosis [8]. Generally, TBIs were classified as mild, moderate, or severe by using the Glasgow Coma Scale (GCS). A TBI with a GCS score of 13–15 is defined as mild TBI, between 9–12 as moderate and 3–8 as severe [37]. An important parameter of the severity of TBI is post- or peri-traumatic amnesia. Post-traumatic amnesia (PTA) of 1–24 h indicates a moderately severe TBI; however, more recent classifications of moderate TBI require post-traumatic amnesia extending beyond 24 h [38,39].

A widely acceptable TBI classification system is the Mayo System which divided TBIs as possible, probable-moderate, and definite moderate-severe [39]. A TBI is classified as probable mild if there is loss of consciousness below 30 min, post-traumatic amnesia for less than 24 h, and there is a depressed, basilar, or linear skull fracture, but with intact dura matter. A TBI is classified as possible if the patient develops blurred vision, confusion, headache, or nausea, and as definite moderate-severe if there is loss of consciousness lasting 30 min or more, post-traumatic amnesia of 24 h or more, or worst full Glasgow Coma Scale score below 13, or if there is death due to this TBI. The Mayo Classification System also requires that all other causes of impaired consciousness should be excluded. If there is additional evidence of brain hematoma, haemorrhage, contusions, or ruptured dura mater, the TBI is classified as moderate-severe [40].

### 3.2. Post-Concussion Syndrome

Post-concussion syndrome (PCS) is a sequela of minor brain injury. Although about 29–90% of patients may experience PCS after a head injury [16,17,18,19,20,21], its etiology is unclear. Despite that no universally accepted definition of PCS exists, it is generally accepted as the development of at least three of the following symptoms: headache, fatigue, irritability, dizziness, and balance issues, affected sleep, poor memory and concentration, and increased sensitivity to light and noise. The symptoms occur shortly after a head impact and could persist for weeks or months. When the symptoms persist for more than six months or one year, the condition is defined as persistent PCS (Table 1). PCS is usually characterized by the absence of objective findings and inconsistencies in presentation [24].

The ICD-10 diagnostic criteria for PCS include a history of traumatic brain injury and the presence of three or more of the following: headache, dizziness, fatigue, irritability, insomnia, concentration or memory disturbance, and intolerance to stress, alcohol, and emotion [42,43].

However, because The American Psychiatric Association’s and Statistical Manual of Mental Disorders, Fifth Edition (DSM-5), defines PCS as a major or mild neurocognitive disorder due to traumatic brain injury and requires evidence of traumatic brain injury with any of the following: loss of consciousness, post-traumatic amnesia, disorientation and confusion, neurological signs such as new onset of seizures, anosmia, or hemiparesis, recent reports suspected that post-concussive consequences could affect the trauma-exposed brain for a longer period of time which lead to the suggestion that long-termed effects of PCS could go beyond neurological traits. Thus, it was shown that the neurocognitive symptoms occur directly after the traumatic brain injury or immediately after regaining consciousness and persist for longer than the acute post-injury period [46]. While searching for evidence regarding the neurocognitive impairment processes occurring post-concussive, it was suggested that complex mechanisms neurodegeneration and neuroinflammation are both involved in this matter. 

### 3.3. Chronic Traumatic Encephalopathy

Chronic Traumatic Encephalopathy (CTE) was initially introduced as “punch drunk” or dementia pugilistica in the early 1900s. It was first described in boxing, where many retired boxers developed dementia at a higher incidence than the general population [10,13,47,48].

The definition of CTE is mainly based on neuropathological changes, and the term traumatic encephalopathy syndrome (TES) refers to the clinical syndrome associated with exposure to repetitive head impacts.

Traumatic encephalopathy syndrome and CTE do not include the acute or post-acute manifestations of a concussion or post-concussion syndrome. 

TES/CTE is classified into probable, possible, and improbable, based on clinical presentation and the pathologic changes [44]. Jordan et al. (2013) proposed that definite TES should include neurologic signs and symptoms in keeping with CTE, including behavioural or cognitive disturbance and motor symptoms [44]. Pathologic confirmation of tau deposition in brain autopsy could also be considered definitory [25]. Probable TES is described as two or more of the following: cognitive and/or behavioural impairment, cerebellar dysfunction, pyramidal tract disease, or extrapyramidal disease. Thus, Jordan et al. (2013), as well as Reams et al. (2016), suggested that the diagnosis could be supported by abnormal neuroimaging findings on positron emission tomography, single-emission tomography, structural magnetic resonance imaging, or diffusion-tensor imaging [26,44]. 

There are multiple clinical diagnostic criteria for TES/CTE for clinically probable and possible TES [25,26,27]. The former requires a history of head trauma exposure, the persistence of symptoms for longer than two years, lack of another diagnosis to otherwise explain the signs and symptoms, and the presence of at least two symptoms, such as speech, mood, or behavioural disturbance, and three signs including ataxia, memory loss, and dysarthria [27]. 

Montenigro et al. (2015) proposed diagnostic criteria which require repetitive head trauma, the persistence of symptoms for longer than one year, and the absence of another neurologic disorder that could otherwise account for the symptoms [27]. Furthermore, one core clinical feature, including cognitive, behavioural, or mood disturbance, and two supportive features, including headache, motor signs, behavioural changes with impulsivity, anxiety, apathy, paranoia, suicidality, progressive decline, or delayed onset, are required for the diagnosis of TES [22,27].

The diagnostic criteria proposed by Reams et al. (2016) persistence of symptoms for longer than two years, the absence of another neurologic disorder, which could more likely account for all the clinical features, history of exposure to head trauma associated with a history of concussion, or subconcussive head injury, history of repetitive head trauma, confirmed progressive course, late symptom onset, and self-reported or observer reported cognitive dysfunction, and cognitive decline confirmed by neuropsychological testing [26]. Reams et al. (2016) also proposed supportive features for the diagnosis, including emotional dysregulation, behavioural changes, and motor disturbance [26].

Based on the clinical presentation, TES/CTE can be further classified into behavioural or mood variant, cognitive variant, mixed variant, or dementia variant, and based on the progression, into progressive type, stable type, unknown or inconsistent type [26].

#### 3.3.1. Staging of CTE

**Figure 1:**
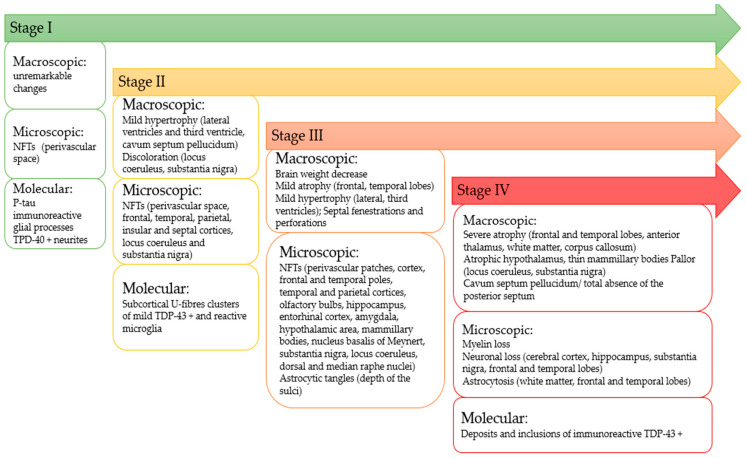
Timeline of CTE staging.

At stage I, the brain is usually macroscopically unremarkable; however, the microscopic examination may show focal deposition of p-tau neurofibrillary tangles and neurites at the perivascular spaces (Figure 1). Occasional glia and p-tau immunoreactive glial processes were also observed [22]. The pathology is mainly confined to the depth of the sulci of the temporal, parietal, frontal, and insular cortices. Occasional p-tau astrocytic tangles can be seen in clusters at the depths of sulci, and neurofibrillary tangles can also be noticed in the locus coeruleus. About 50% of brains with stage I CTE may show infrequent TDP-43 positive neurites. At this stage, reactive microglia with axonal swelling and distorted profiles are seen in the subcortical U-fibres [22].

At stage II, macroscopic examination shows a mild enlargement of the frontal horns of the lateral ventricles and third ventricle, cavum septum pellucidum, and discoloration of the locus coeruleus and substantia nigra. Microscopic examination reveals multiple foci of perivascular p-tau neurofibrillary neurites and tangles and neurites at the frontal, temporal, parietal, insular, and septal cortices, consisting of tangles or pre-tangles, neurites, and glia in addition to p-tau neurofibrillary tangles. The locus coeruleus and the substantia nigra can also exhibit neurofibrillary tangles. Additionally, mild TDP-43 pathology and clusters of reactive microglia are found in clusters in the subcortical U-fibres. [22].

Stage III brains show macroscopic and structural changes consisting of decreased brain weight, mild atrophy at the frontal and temporal lobes, and expansion of the lateral and third ventricles. Septal abnormalities with cavum septum pellucidum, septal fenestrations, and perforations are also seen in 50% of cases. Additional macroscopic changes may consist of pallor of the substantia nigra and the locus coeruleus, atrophy of thalamus, hypothalamus, and mammillary bodies, and thinning of the corpus callosum. Microscopic examination reveals perivascular patches of neurofibrillary tangles, neurites, and astrocytic tangles at the depths of the sulci. At this stage, linear arrays of neurofibrillary tangles and neurites can also be seen in the superficial laminae of the cortex. Neurofibrillary tangles are found in the frontal, and temporal poles, the temporal and parietal cortices, in the olfactory bulbs, the hippocampus, the entorhinal cortex, and the amygdala, the hypothalamic area and mammillary bodies, the nucleus basalis of Meynert, substantia nigra, the locus coeruleus and dorsal and median raphe nuclei. About 30% of cases also exhibit occasional neurofibrillary tangles [22].

Brain weight is significantly decreased at stage IV. There is noticeable atrophy of the frontal and temporal lobes, the anterior thalamus. Generalized cerebral atrophy and diffuse white matter atrophy, and atrophy of the corpus callosum are also observed. The hypothalamic area is usually atrophic, and the mammillary bodies can also be thin. Cavum septum pellucidum or total absence of the posterior septum with the pallor of the locus coeruleus and substantia nigra are also commonly found. Microscopically there is a prevalent myelin loss, neuronal loss in the cerebral cortex, the hippocampus, and the substantia nigra, and astrocytosis of the white matter. Extensive neuronal loss and astrocytosis with microvacuolation of layer II may be seen in the frontal and temporal lobes. P-tau positive tangles are distributed all through the cerebral cortex, thalamus and hypothalamus, mammillary bodies, basal ganglia, brainstem, cerebellar dentate nucleus, and spinal cord. Tau pathology may also involve the cerebellum, including Purkinje cells, the dentate nucleus, and the granular layer. Examination of the white matter of the anterior cerebellar vermis may reveal irregular neurites. Deposits and inclusions immunopositive for TDP-43 are also commonly seen [22,49].

#### 3.3.2. Neurophysiological Pathological Description

Macroscopic pathology. In the early stages of CTE, the brain is usually unremarkable; however, macroscopic examination can rarely reveal cavum septum pellucidum and mild enlargement of the frontal and temporal horns of the lateral ventricles, along with slightly prominent perivascular spaces in the white matter of the temporal lobe. At the advanced stages of the disease, the brain weight is reduced, and there is prominent grey and white matter atrophy, typically more severe in the frontal and anterior temporal lobes. Cavum septum pellucidum, and/or septal fenestrations, additional enlargement of the lateral and third ventricles, thinning of the isthmus of the septum corpus callosum, thalamic and hypothalamic atrophy and atrophy of the mammillary bodies, and depigmentation of the substantial nigra and locus coeruleus are usually seen. The cerebellum is usually unremarkable; however, the initial reports of CTE described in boxers described macroscopic changes of the cerebellum [22].

Microscopic pathology. Chronic traumatic encephalopathy is a tauopathy that could be diagnosed with precision using post-mortem neuropathological examination. The pathognomonic hallmark of CTE is the deposition of phosphorylated tau (p-tau) protein in the forms of neurites or neurofibrillary tangles in the neocortex, typically placed alongside small blood vessels at the depths of the sulci. The neurofibrillary tangles follow the small cortical vessels forming linear accumulations that extend from the surface of the brain to the deepest layers of the grey matter. They can also be seen in the forms of clusters of neurofibrillary tangles, pre-tangles, and thread-like neurites around small arterioles. Focal subpial p-tau astrocytes and occasional p-tau glia around vessels can be seen in the advanced stages [22].

However, the mechanisms behind CTE are not fully known. It is believed that the axonal injury following a head impact can lead to phosphorylation of tau protein and subsequently to tau deposition [50,51]. It is believed that the acceleration-deceleration type of injury causes abnormal phosphorylation of tau-protein, which then will become misfolded, aggregated, and cleaved [51]. It is also thought that the hyperphosphorylated tau protein is related to a prion-like propagation in TBI; it is also plausible that hyperphosphorylated tau deposition promotes the accumulation of other aggregate-prone proteins such as amyloid and TAR DNA-binding protein 43 (TDP-43) [17,18]. Interestingly, the inoculation of brain homogenates from mice with TBI into the hippocampus and cerebral cortex of wild-type mice seem to enhance the propagation of tau and the development of memory impairment. The same effects were noticed when brain homogenates obtained contralateral to the side of TBI were used, supporting the prion-like propagation mechanism of tau protein [52]. Severe axonal loss and axonal swellings and varicosities are found in the advanced stages of CTE. Axonal injury is thought to be related to the initiation of p-tau pathology [18,50]. Damage to axons due to repetitive brain injury may cause changes in membrane permeability and ionic shifts resulting in a large influx of calcium and subsequent release of caspases and calpains, which would trigger tau phosphorylation [53]. A cascade of events then involving activation of microglia and release of toxic cytokines, chemokines, immune mediators, and excitotoxins like glutamate, aspartate, and quinolinic acid leads to additional hyperphosphorylation of tau protein and dysfunction of microtubules and deposition of neurofibrillary tangles [51,53,54].

The neuropathological criteria for the diagnosis of chronic traumatic encephalopathy require deposition of perivascular foci of p-tau immunoreactive pre-tangles and dot-like and thread-like neurites in the neocortex, irregular distribution of p-tau immunoreactive neurofibrillary tangles, astrocytic tangles, and dot-like and thread-like neurites at the depths of cerebral sulci, often alongside penetrating blood vessels, and neurofibrillary tangles in the crests of the cerebral cortex located preferentially in the superficial layers II and III. Supportive features include the presence of clusters of subpial astrocytic tangles in the cerebral cortex, most prominent at the sulcal depths, and subependymal astrocytic tangles in the periventricular regions of the lateral ventricles periaqueductal grey matter and lateral brainstem [22].

## 4. Clinical Biomarkers 

### 4.1. Neuroimaging

#### 4.1.1. Magnetic Resonance Imaging (MRI)

Severe traumatic brain injury can result in diffuse white matter injury, focal contusions, or hemorrhages that can be seen on conventional MRI or CT (Table 2). Several studies on the white matter integrity using diffusion tensor imaging (DTI) support the assertion that repetitive asymptomatic head trauma concussion and mild traumatic brain injuries result in damage to cortical and subcortical microstructures despite observable findings on conventional MRI being absent [55,56]. The genu and the body of the corpus callosum most consistently show evidence of microstructural changes associated with head trauma [57,58]. It is thought that accumulated exposure to repetitive head trauma would lead to brain tissue loss and measurable differences in brain volume compared to healthy individuals. The impact of RMHI has primarily been studied in boxing athletes and professional American football players; however, most studies had small samples. The main differences that have been described in symptomatic former professional head impact-prone sports players are reduced volumes of the amygdala, hippocampus, cingulate gyrus, fronto-insular and anterior-temporal volumes [59,60,61]. Steeper hippocampal volume loss and lower thalamic volumes have also been associated with earlier age of starting American football participation [60]. Another study on 476 professional boxers and martial art athletes versus 63 controls found decreased thalamic and callosal volumes in the athletes’ group [61].

One study on retired professional soccer players revealed reduced cortical thickness in the occipital and inferior parietal temporal lobes [62].

A study on military personnel found the increased thickness of the cerebral cortex in occipital, frontal, temporal, parietal, praecuneus, and cingulate cortices compared to controls [63]. The authors concluded that these findings could reflect glial scaring or changes in cortical myelination at the grey-white matter junction.

White matter changes, usually close to grey-white matter junction, and predominantly into the frontal lobes, along with decreased susceptibility-weighted imaging signal which can represent trauma-related microbleeds have also been described; however, these findings seem to be non-specific [64].

Cavum septum pellucidum is one of the most described findings and was first recognized by Forster in 1933 in a patient who suffered a brain injury and later died. It was then described in professional fighters and professional football players [61,65,66]. Cavum septum pellucidum and longer Septum Pellucidum were more frequently described in American football players with cognitive impairment than controls [67]. It is unclear whether these changes onto the Septum Pellucidum represent the consequence of repetitive traumatic brain injuries. 

#### 4.1.2. Diffusion MRI

Herweh et al. (2016) performed a DTI study on 31 amateur boxers and 31 control individuals, and they reported significantly reduced fractional anisotropy (FA) in the boxers group [68]. Another survey of 10 football players with no apparent history of clinically defined concussions but a history of more than 430 head impacts per year and 5 controls found greater changes in FA and the average rate of diffusion in all directions in the football players group [64]. The authors concluded that repetitive head injuries, even the sub-concussive ones, can result in white matter changes that persist after six months of contact-free rest [64]. Additional markers that can differentiate between axonal and myelin injury in DTI are the radial diffusivity (RD) and the axial diffusivity (AD). Multani et al. (2016) investigated the AD differences between retired Canadian football players and age-matched controls and they reported a significant increase in AD, in the footballers’ groups [67]. Koerte et al., 2012 investigated the white matter integrity in soccer players without clinically defined concussions, compared to swimmers and reported increased RD in football players across different white matter structures and higher AD in the corpus callosum [69].

The neurite orientation dispersion and density imaging (NODDI) is a DTI technique that can show changes of axons and dendrites and can also provide information on neurite density and orientation [85]. Using NODDI in athletes following a sport-related concussion, Churchill et al., 2019 found that decreases in fractional anisotropy and increases in axial and radial diffusivity were associated with reduced intraneuritic water volume [70]. They also reported a positive correlation between the severity of symptoms and changes in fractional diffusivity axial and radial diffusivity [69].

#### 4.1.3. Functional MRI

Abnormalities in functional MRI (fMRI) in patients with mild TBIs have been reported in multiple studies [71,72,73,74]. Repetitive head injury is related to acute and long-term changes, while irregularities in the default mode network and other white matter changes have been described [71,72,73]. Han et al. (2016) found significant changes in connectivity in the default, dorsal attention, and frontoparietal control networks [73], and Nordin et al., 2016 revealed a significant correlation between mental fatigue and functional connectivity in patients with a history of mild TBIs [74]. Significant reductions in cerebrovascular reactivity have also been reported in mean global, grey and white matter areas in patients with chronic TBI [75].

#### 4.1.4. Magnetic Spectroscopy (MRS)

MRS measures human brain metabolism in vivo. Alosco et al. (2019) on an MRS study in 77 symptomatic retired NFL players, reported a positive correlation between behavioural/mood symptoms and neurochemicals related to neuroinflammation [76]. They described a positive correlation between accumulative head impacts and lower parietal white matter creatine levels. In another study, Lin et al. (2015) found significantly higher values in glutamine/glutamate, choline, fucosylated molecules, and phenylalanine in five former professional athletes compared to control individuals [77]. 

#### 4.1.5. Susceptibility Weighted Imaging (SWI)

SWI is sensitive to venous blood and can detect hemorrhage or microbleeds in traumatic brain injuries. Studies in patients with a history of mild TBIs have shown a correlation between SWI findings and cognitive outcome [78].

#### 4.1.6. Positron Emission Tomography (PET)—Metabolic and Molecular Neuroimaging

FDG-PET. 2-deoxy-2-(18F) fluorodeoxyglucose can provide in vivo evidence of the severity and distribution of brain changes presumably representing altered synaptic activity. Former boxers with a history of repetitive brain injury revealed hypometabolism in multiple brain regions, including posterior cingulate, bilateral frontal lobes, parieto-occipital cortex, and cerebellum. The findings were very inconsistent between studies [79,80]. At least one study on military veterans showed lower cerebellar metabolism correlated with a higher number of prior blast exposures [81]. One study in American football players revealed significantly lower frontotemporal metabolism than controls [82].

Aβ-PET and Tau-PET. Although Aβ deposition is a common co-pathology in advanced CTE cases [83], it occurs at an accelerated rate and predominantly affects the depths of cortical sulci [84]. Many tracers, including FDDNP, flortaucipir, and FTP, have been developed to detect tau deposition in CTE; however, the sensitivity and specificity of most of them remain low; thus, their use is limited [85,86,87,88,89].

### 4.2. Fluid Biomarkers 

Although the pathophysiology and the underlying mechanisms of PCS and the other discussed concussion, TBI, and RMHI are not yet clearly understood. Neuroimaging could offer viable diagnostic solutions; fluid biomarkers could still be a good alternative, considering that molecular biomarkers also have predictive value and could show many pathological molecular features which occur before other visible/detectable symptoms. In this way, fluid biomarkers could be of crucial interest in the context of predicting the neurodegeneration processes to which predisposing risk repetitive brain injuries are contributed.

Thus, recent studies on the relevant body fluids dynamics during and following head injuries showed that several molecules occurring in blood, cerebrospinal fluid (CSF), saliva, and even urine recorded specific changes of suggestive diagnostic value. In this way, altered plasma tau concentrations are reported to be related to the persistent post-concussion syndrome in military personnel with a history of TBIs [90], with the authors of the study concluding that there might be an association between tau or axonal injury and PCS. Additional recent studies on professional athletes with PCS and matched controls showed increased NFL concentration, astroglia activation, and Aβ peptide dysmetabolism in the brain [91,92,93], however further studies are required. A recent meta-analysis showed significantly increased serum light neurofilament chain (NFL) levels in all patients with a history of concussion compared to controls. That sports-related concussion was specifically associated with higher levels of NFL, marking the potential of NfL levels as a biomarker in mild TBI and head impacts [94].

A study on 96 symptomatic retired National Football League players and 25 age-matched controls showed that plasma-Tau was positively associated with an estimate of cumulative exposure to repetitive head injuries [95]; however, no significant difference was found between the groups of the study.

Another study on 78 former NFL players and 16 control individuals reported findings of tau-positive exosomes in plasma in the players’ group [96], a result that was associated with lower scores on tests of memory and decreased psychomotor speed. Increased exosomal tau has been recently described in military personnel with a history of mild TBI and persistent post-concussive symptoms [97,98]. Furthermore, as exosomal tau is also relevant in neuroinflammation, several studies showed that some neuroinflammatory biomarkers in both PCS and CTE in blood and CSF registered specific significant changes [95,96,98,99,100,101,102], suggesting that the neuroinflammatory component of post-concussive processes remained active a longer period following the traumatic event. In this way, IL6, IL8, and TNF-α [103], which were all significantly increased following TBI, are also involved in progressive neurodegeneration [104,105,106], suggesting that neuroinflammation biomarkers should be considered as potentially relevant while evaluating the neurodegenerative risk in concussed patients. However, extensive studies on the specific features of neuroinflammation correlation to neurodegeneration in the TBI context are needed to elucidate the biomarker evaluation aim and approach in this matter. 

On the other hand, in profession-related CTE patients, the pattern of molecular changes could be seen mainly in CSF due to neuroinflammatory processes, such as microglial activation. In this sense, Alosco et al. (2016) showed that CSF levels of post-concussive traditionally altered biomarkers, such as total tau, p-tau, and Aβ peptide 1-42 were not significantly different in a group of 68 former NFL players, as compared to 21 controls, while the microglial activation biomarker sTREM2 was significantly higher. Moreover, it was shown that increased sTREM2 levels are correlated with increased total tau concentrations, suggesting the potential link between neuronal injury and microglial activation in CTE [99] (Table 3). However, other studies showed consistent changes in other blood and CSF brain injury-related biomarkers in CTE patients [95,96]. In addition, Cheng et al. (2019) [107] reported that several Alzheimer’s disease-relevant salivary biomarkers used to evaluate cellular membrane damage showed similar modification patterns in CTE patients. 

**Figure 2:**
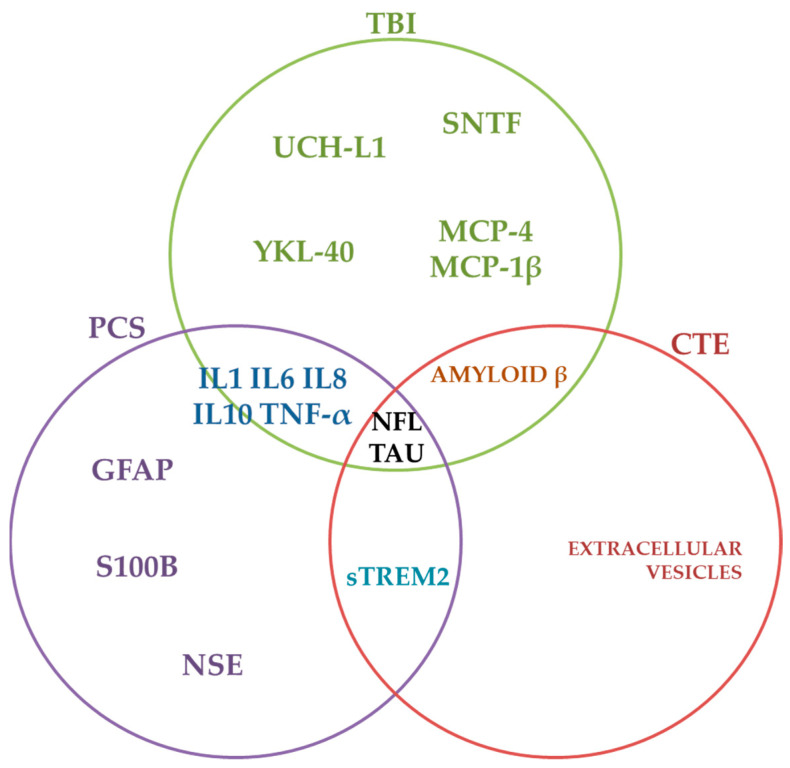
Summative schematization of fluid biomarkers in concussion-related disorders.

Considering these aspects, the future perspectives in neurodegeneration processes initiation during or following predisposing events, such as concussion, TBI, RMHI, and subsequent short or long-termed consequences, include further analysis of molecular processes shifting in PCS and CTE. Also, the fact that neurodegeneration and neuroinflammation specific biomarkers are relevant in PCS and CTE (Figure 2) context could suggest that the risk for neurodegeneration initiation in these conditions could be regarded as important, and further efforts are needed to specifically evaluate and be considered in neurology preventive approaches.

## 5. Concluding Remarks and Future Perspectives

Traumatic brain injury (TBI) represents one of the main contributors to death and disability worldwide. While TBI following singular concussion incidents does not necessarily imply long-term consequences (such as chronic traumatic encephalopathy, TES/CTE), the repeated mild head injuries as occurring in sports and professions at risk could cumulatively lead to an increased risk for chronic brain injury-associated nervous system impairments and for subsequent impairments, such as neurodegeneration. 

Both post-concussive syndrome (short-termed consequence of TBI) and CTE (a long-termed consequence of repeated TBI) have widespread implications for athletes, military service members, victims of abuse, and patients with epilepsy who have multiple brain injuries. Numerous studies have been performed to identify imaging and fluid biomarkers that could assist in the diagnosis and would probably lead to early intervention; however, the outcomes of most of them are heterogeneous. Despite that, there are several shortcomings regarding the diagnostic possibilities and predicted outcomes considering that CTE could greatly increase the risk for neurodegeneration occurrence, as compared to PCS. In this way, further studies in high-risk populations are required to establish certain, preferably non-invasive biomarkers for CTE that could be used during the patient’s life for prevention, early diagnostic, risk assessment, outcome prediction, or management performance.

## Figures and Tables

**Table 1:** Classification systems of head injury syndromes and diagnostic criteria.

Disorder	Classification System	Stages	Diagnostic Criteria
Concussion [36]	Nelson Grading system	• Grade 0	not stunned or dazed; headache, difficult concentration;
		• Grade 1	stunned or dazed; no LOC or PTA; sensorium recovery < 1 min;
		• Grade 2	headache; sensorium recovery > 1 min; no LOC; tinnitus, amnesia, irritability, hyperexcitability, confusion, dizziness;
		• Grade 3	LOC < 1 min; no coma; grade 2 symptoms during recovery;
		• Grade 4	LOC > 1 min; no coma; demonstrates grade 2 symptoms during recovery
	Ommaya grading system	• Grade 1	Confusion; no PTA;
		• Grade 2	PTA without coma;
		• Grade 3	Coma < 6 h
		• Grade 4	Coma = 6–24 h
		• Grade 5	Comas > 24 h
		• Grade 6	Coma, death within 24 h
	Jordan grading system	• Grade 1	Confusion; no PTA, LOC;
		• Grade 2	Confusion; PTA < 24 h; no LOC;
		• Grade 3	LOC (altered level of consciousness < 2–3 min); PTA < 24 h;
		• Grade 4	LOC (altered level of consciousness > 2–3 min);
	Torg grading system	• Grade 1	Tinnitus; short-term confusion; dazed; no PTA;
		• Grade 2	PTA; vertigo; no LOC;
		• Grade 3	PTA retrograde; vertigo; no LOC;
		• Grade 4	Immediate transient LOC;
		• Grade 5	Paralytic coma; cardiorespiratory arrest;
		• Grade 6	Death
	Colorado Medical Society guidelines	• Mild	Confusion; no PTA, LOC;
		• Moderate	Confusion; PTA; no LOC;
		• Severe	LOC.
	Cantu grading system	• Mild	No LOC; PTA < 30 min;
		• Moderate	LOC < 5 min; PTA > 30 min;
		• Severe	LOC > 5 min or PTA > 24 h.
	Roberts grading system	• Bell ringer	No LOC, PTA; recovery < 10 min;
		• Mild	No LOC; PTA < 30 min; recovery > 10 min;
		• Moderate	LOC < 5 min; PTA > 30 min;
		• Severe	LOC > 5 min; PTA > 24 h.
	Kelly and Rosenberg grading system	• Mild	Transient confusion; no LOC; symptoms resolve in < 15 min;
		• Moderate	Transient confusion; no LOC; symptoms last > 15 min;
		• Severe	Brief or prolonged LOC.
Traumatic brain injury (TBI)	Glasgow Coma Scale (GCS) [37]	• Mild	CGS score = 13–15
		• Moderate	CGS score = 9–12
		• Severe	CGS score = 3–8
	PTA Mississippi intervals [38,39]	• Moderate	PTA 1–24 h
		• Severe	PTA > 24 h
	Mayo system [40]	• Possible	Blurred vision, confusion (mental state changes), dazed, dizziness, focal neurologic symptoms, headache, nausea
		• Probable—mild	Loss of consciousness < 30 min, PTA < 24 h, skull fracture (dura intact)
		• Definite—moderate/severe	Loss of consciousness > 30 min, PTA > 24 h, CGS score (first 24 h) < 13, skull fracture (with hematoma, hemorrhage, concussion, or brain stem injury), death
	Glasgow Outcome Scale [33]	• Dead	
		• Vegetative state	Lack of function in the cerebral cortex, although structurally intact
		• Severe disability	Conscious, total dependency on caregiver (severe physical and mental disability)
		• Moderate disability	Independent, but disabled (physical and mental disability)
		• Good recovery	Minor physical and mental disability
	Russell and Smith’s classification system [41]	• Severe	PTA = 1–7 days
		• Very severe	PTA = +7 days
	Nakase–Richardson classification system [38]	• Moderate	PTA = 0–14 days
		• Moderately severe	PTA = 15–28 days
		• Severe	PTA = 29–70 days
Post-concussion syndrome (PCS)	Ontario Neurotrauma Foundation [42,43]		Symptoms according to ICD10 or DSM-V
		• Minor	Symptoms duration = 1–6 months
		• Persistent	Symptoms duration > 6 months
Traumatic encephalopathy syndrome (TES)/Chronic Traumatic Encephalopathy (CTE)	Jordan classification system [44]	• Improbable CTE	Pathophysiological process unrelated to brain trauma;
		• Possible CTE	CTE clinical description also seen in other neuropathologies;
		• Probable CTE	Cognitive and/or behavioral impairment; morpho-functional changes in central nervous system;
		• Definite CTE	CTE clinical presentation and pathological confirmation.
	Montenigro clasiffication system [27]	• Behavioural/mood variant	Behavioural and mood features;
		• Cognitive variant	Cognitive impairment;
		• Mixed variant	Both behavioural and cognitive impairments;
		• Dementia variant	Progressive cognitive decline dependent or independent of Alzheimer’s disease diagnostic.
	Omalu neuropathological classification [45]	• Phenotype I	+NFTs and neuritic threads (cerebral cortex and brainstem) −diffuse amyloid plaques
		• Phenotype II	+NFTs and neuritic threads (cerebral cortex and brainstem)
		• Phenotype III	−diffuse amyloid plaques (cerebral cortex)
		• Phenotype IV	+NFTs and neuritic threads (brainstem) −diffuse amyloid plaques −NFTs and neuritic threads −diffuse amyloid plaques

**Table 2:** Neuroimaging biomarkers for traumatic brain injury following repeated head impacts in PCS and CTE.

Method	Disorder	Observable Features	Clinical Studies
Magnetic Resonance Imaging (MRI)	PCS, CTE	White matter injury, focal concussions, haemorrhages	• 86 symptomatic former NFL players—the decreased amygdala, hippocampus, and cingulate gyrus volumes [57,60] • 33 male Canadian football players—changes in the microstructural integrity of the white matter (anterior and posterior regions of the corpus callosum) [58] • 476 active and retired professional fighters and 63 controls—the presence of cavum septum pellucidum and cavum vergae, lower brain volumes in the supratentorium [61] • 15 former male professional soccer players and 15 male, age-matched former professional non-contact sport athletes-right inferolateral-parietal, temporal, and occipital cortex thinning [62] • 20 current or previous military or civilian law enforcement breachers and 14 controls—increased cortical thickness (occipital lobes) [63] • 10 Division III college football players and five non-athlete controls-changes in fractional anisotropy and mean diffusivity (white matter) [64] • 499 fighters (boxers, mixed martial artists, and martial artists) and 62 controls-increased prevalence of cavum septum pellucidum and cerebral microhemorrhages among fighters [65] • 72 symptomatic former professional football players and 14 former professional noncontact sports athletes-presence of cavum septum pellucidum [66]
Diffusion tensor imaging (DTI)	TBI, RMHI	Asymptomatic head trauma concussion, mild traumatic brain injuries (cortical and subcortical microstructures)	• 18 retired professional football players and 17 healthy controls’chronic axonal degeneration (superior longitudinal fasciculus, corticospinal tract, and anterior thalamic radiations) [67]
Diffusion tensor imaging	TBI, RMHI, CTE	Random Brownian motion of water molecules within a voxel of tissue, cellular swelling, grey matter status (cerebral cortex nuclei)	• 31 amateur boxers and 31 control individuals—reduced fractional anisotropy, increased diffusivity along central white matter tracts [68] • 12 soccer players and 11 swimmers—increased radial diffusivity and axial diffusivity in soccer players [69] • 10 Division III college football players and five non-athlete controls—up to 6 months persistent white matter changes [64] • 18 retired professional football players and 17 healthy controls—changes in axial diffusivity [67]
Neurite orientation dispersion and density imaging (NODDI)	PCS	Acute alterations in microstructure (neurite density and orientation, axons and dendrites)	• 31 concussed athletes and 27 matched controls - reductions in fractional anisotropy and increased axial and radial diffusivity, increased neurite dispersion [70]
Functional MRI	TBI	brain activity (changes associated with blood flow)	• 15 varsity level college students who sustained a sports-related concussion and 15 age and sex-matched controls—increased activation of prefrontal area (BA46, BA10) and left inferior parietal (supramarginal) gyrus (BA40) [71] • Seven college athletes and 11 healthy controls—increased cerebrovascular reactivity in all evaluated regions and independently in anterior cingulate and the right thalamus and increased focal connectivity in left and right hippocampus, precuneus and ventromedial prefrontal cortex [72] • 40 chronic TBI individuals and 17 healthy individuals—reduced connectivity in TBI individuals in the default mode network, dorsal attention network, and frontoparietal control network and in between interactions [73] • 10 mild TBI patients and 10 healthy controls—decreased functional connectivity networks in thalamus, dorsal anterior cingulate and medial frontal gyri [74] • 27 TBI patients and 15 healthy controls—asymmetry in cerebrovascular reactivity and cerebral blood flow maps in TBI (multifocal pattern of deficits) [75]
Magnetic Spectroscopy (MRS)	TBI, RMHI	Human brain metabolism in vivo	• 77 symptomatic former NFL players and 23 asymptomatic individuals without a head trauma history—significantly lower N-acetyl aspartate level in the parietal white matter [76] • Five former professional male athletes and five healthy men—increased levels of glutamine/glutamate, choline, fucosylated molecules, and phenylalanine [77]
Susceptibility weighted imaging (SWI)	TBI	Hemorrhage, microbleeds in the brain	• 106 children with TBI and 43 healthy controls—increased number and volume of lesions in TBI group, predominantly in the frontal, extra frontal, deep grey, and cerebellum regions [78]
Positron Emission Tomography (PET) FDG-PET Tau-PET Aβ-PET	TBI, CTE	Severity and distribution of brain changes (altered synaptic activity)	• 19 boxers and 17 controls—altered activity in the posterior cingulate cortex, parieto-occipital, frontal lobes (Broca’s area) bilaterally, and the cerebellum [79] • Five retired professional boxers and four age-matched controls—neuronal deficits in angular gyrus and temporal cortical regions [80] • 33 veterans with mild TBI—lower cerebellar metabolism [81] • 11 Traumatic Encephalopathy Syndrome patients—elevated tau-PET binding, lower gray matter volumes in frontotemporal areas [82] • 202 football players—p-tau clusters in medial temporal lobe, cerebral cortex, diencephalon, and brain stem [83] • 114 CTE-diagnosed deceased athletes and military veterans—diffuse or neuritic Aβ deposition present in 52 % of CTE subjects [84]

**Table 3:** Fluid biomarkers for traumatic brain injury following repeated head impacts.

Biomarker	Disorder	Observable Features	Clinical Studies
Serum neurofilament light polypeptide (NFL)	TBI	axonal injury	• 19 American football athletes and 19 swim athletes—increased levels of NFL in football athletes, not normalizing even after nine months following TBI [108]
Serum S-100 calcium-binding protein B and neuron-specific enolase (NSE)	PCS	astroglial injury general neuronal injury	• 47 preseason and 28 PCS professional ice hockey players—increased S100B in PCS, no changes in NSE levels; levels of S-100B and NSE also increasing following physical effort [109] • 44 amateur boxers and 23 healthy controls—increased serum NSE in amateur boxers, no changes in S100B levels [110]
Serum neurofilament H and SNTF	TBI	stretch injury of neuronal axons	• Nine TBI and three controls—increased SNTF and NFH in sera of TBI patients receiving surgical brain pressure release [111] • 28 concussed and 45 non-concussed professional ice hockey players—diagnostic accuracy increased levels of SNTF in concussed athletes [112]
Serum GFAP	TBI	astrocytic response to neuronal damage	• 215 acute TBI patients—increased serum GFAP levels following acute TBI [113]
Serum IL6, IL8 and TNF-α	TBI	neuroinflammation	• 24 TBI patients—increased serum IL6, IL8, and TNF- α in possible correlation with poor outcome and subsequent additional insults (brain damage) [103]
Exosomal tau	TBI	neuronal loss and neurodegeneration	• 98 veterans with mild TBI with PTA or LOC, 52 with mild TBI without PTA or LOC and 45 without TBI—increased plasma and exosomal tau, p-tau significantly correlated to post-concussive symptoms [98]
	PCS		• 20 current or previous military or civilian law enforcement breachers ad 14 controls—neuronal-derived extracellular vesicles (serum) tau levels increased and correlated Neurobehavioral Symptom Inventory score, in experienced breachers [100] • 42 PCS military personnel and 22 without PCS—increased concentrations of exosomal tau, Aβ42, and IL-10 [95]
	CTE		• 78 former National Football League players and 16 controls—the increased presence of tau-positive extracellular vesicles in former NFL players, as compared to controls [96]
Plasma total tau	PCS	axonal injury	• 70 participants with self-reported TBI compared with the 28 controls—increased plasmatic total tau levels [90] • 47 preseason and 28 PCS professional ice hockey players—increased PCS levels, as compared to preseason evaluation [109]
	RHI		• 96 former NFL players and 25 same-age controls - total-tau plasma concentration ≥ 3.56 pg/mL was specific to repetitive head impact individuals [97]
Plasma UCH-L1, GFAP, Tau	TBI	neuronal damage	• 27 TBI and six controls—increased UCH-L1 in oen TBI patient with abnormal CT scan [114] • 264 contact sport and 138 non-contact sport controls—increased plasma UCH-L1 levels in concussed athletes [115]
Plasmatic calpain-cleaved SNTF	TBI	acute brain damage, neurodegeneration	• 38 participants—increased plasma calpain-cleaved SNTF in TBI and some orthopedic cases, as compared to uninjured controls [116]
Plasma MCP-4 and MCP-1β	TBI	neuroinflammation	• 43 TBI athletes and 102 control athletes—increased blood levels of MCP-4 and MIP-1β [117]
Plasma IL1 and IL6	TBI	neuroinflammation	• 37 severe TBI patients—increased CSF IL1 and IL6 levels in correlation to TBI severity (Glasgow Outcome Scale) [101]
Plasma IL10	TBI	neuroinflammation	• 82 severe head trauma patients, 39 multiple injuries patients, and 37 healthy donors—increased systemic levels of IL10 following multiple injuries, without possibility to discriminate between head and non-head trauma [102]
CSF GFAP, YKL-40, and amyloid β40, β42	PCS	astroglia injury	• 28 professional athletes with PCS and 19 controls—increased glial fibrillary acidic protein (GFAP) and YKL-40, lower Aβ40 and Aβ42 levels [91]
CSF NF light protein, amyloid β	PCS	axonal injury	• 16 ice hockey players with PCS and 15 control individuals—increased NF light protein and decreased amyloid β CSF levels [92]
CSF total tau level	TBI	axonal injury	• 68 former NFL players and 21 controls—higher CSF t-tau levels correlated with cumulative head impact index in NFL players [99]
CSF sTREM2	TBI	microglial activation	• 68 former NFL players and 21 controls—increased sTREM2 levels in repeatedly concussed individuals, significantly associated with increased CSF T-tau levels [99]
CSF IL1 and IL6	TBI	neuroinflammation	• 37 severe TBI patients—increased CSF IL1 and IL6 levels in correlation to TBI severity (Glasgow Outcome Scale) [101]
Salivary extracelluar vesicles	TBI/CTE	cell membrane damage	• 31 concussion trauma patients and 23 controls—many Alzheimer’s disease relevant salivary biomarkers isolated from extracellular vesicles were found to be expressed in concussed patients [104]

## References

[1] Rubiano A.M., Carney N., Chesnut R., Puyana J.C. (2015). Global neurotrauma research challenges and opportunities. Nature.

[2] Dewan M.C., Rattani A., Gupta S., Baticulon R.E., Hung Y., Punchak M., Agrawal A., Adeleye A.O., Shrime M.G., Rubiano A.M. (2019). Estimating the global incidence of traumatic brain injury. J. Neurosurg. JNS.

[3] Bazarian J.J., Wong T., Harris M., Leahey N., Mookerjee S., Dombovy M. (1999). Epidemiology and predictors of post-concussive syndrome after minor head injury in an emergency population. Brain Inj..

[4] Sharp D.J., Jenkins P.O. (2015). Concussion is confusing us all. Pract. Neurol..

[5] Kaur P., Sharma S. (2018). Recent Advances in Pathophysiology of Traumatic Brain Injury. Curr. Neuropharmacol..

[6] Langer L.K., Alavinia S.M., Lawrence D.W., Munce S.E.P., Kam A., Tam A., Ruttan L., Comper. P., Bayley M.T. (2021). Prediction of risk of prolonged post-concussion symptoms: Derivation and validation of the TRICORDRR (Toronto Rehabilitation Institute Concussion Outcome Determination and Rehab Recommendations) score. PLoS Med..

[7] Permenter C.M., Fernández-de Thomas R.J., Sherman A. (2022). Postconcussive Syndrome. StatPearls [Internet].

[8] Saatman K., Duhaime A., Bullock R., Maas A., Valadka A. (2008). Classification of Traumatic Brain Injury for Targeted Therapies. J. Neurotrauma.

[9] McAllister T., McCrea M. (2017). Long-Term Cognitive and Neuropsychiatric Consequences of Repetitive Concussion and Head-Impact Exposure. J. Athl. Train..

[10] Roberts G.W., Allsop D., Bruton C. (1990). The occult aftermath of boxing. J. Neurol. Neurosurg. Psychiatry.

[11] Omalu B.I., DeKosky S.T., Minster R.L., Kamboh M.I., Hamilton R.L., Wecht C.H. (2005). Chronic traumatic encephalopathy in a National Football League player. Neurosurgery.

[12] Omalu B.I., DeKosky S.T., Hamilton R.L., Minster R.L., Kamboh M.I., Shakir A.M., Wecht C.H. (2006). Chronic traumatic encephalopathy in a national football league player: Part II. Neurosurgery.

[13] Omalu B.I., Fitzsimmons R.P., Hammers J., Bailes J. (2010). Chronic traumatic encephalopathy in a professional American wrestler. J. Forensic Nurs..

[14] Omalu B.I., Hamilton R.L., Kamboh M.I., DeKosky S.T., Bailes J. (2010). Chronic traumatic encephalopathy (CTE) in a National Football League Player: Case report and emerging medicolegal practice questions. J. Forensic Nurs..

[15] Omalu B.I., Bailes J., Hammers J.L., Fitzsimmons R.P. (2010). Chronic traumatic encephalopathy, suicides and parasuicides in professional American athletes: The role of the forensic pathologist. Am. J. Forensic Med. Pathol..

[16] McKee A.C., Cantu R.C., Nowinski C.J., Hedley-Whyte E.T., Gavett B.E., Budson A.E., Santini V.E., Lee H.-S., Kubilus C.A., Stern R.A. (2009). Chronic traumatic encephalopathy in athletes: Progressive tauopathy after repetitive head injury. J. Neuropathol. Exp. Neurol..

[17] McKee A.C., Gavett B.E., Stern R.A., Nowinski C.J., Cantu R.C., Kowall N.W., Perl D.P., Hedley-Whyte E.T., Price B., Sullivan C. (2010). TDP-43 proteinopathy and motor neuron disease in chronic traumatic encephalopathy. J. Neuropathol. Exp. Neurol..

[18] McKee A.C., Stern R.A., Nowinski C.J., Stein T.D., Alvarez V.E., Daneshvar D.H., Lee H.S., Wojtowicz S.M., Hall G., Baugh C.M. (2013). The spectrum of disease in chronic traumatic encephalopathy. Brain.

[19] Hof P.R., Knabe R., Bovier P., Bouras C. (1991). Neuropathological observations in a case of autism presenting with self-injury behavior. Acta Neuropathol..

[20] Geddes J.F., Vowles G.H., Nicoll J.A., Revesz T. (1999). Neuronal cytoskeletal changes are an early consequence of repetitive head injury. Acta Neuropathol..

[21] Goldstein L.E., Fisher A.M., Tagge C.A., Zhang X.L., Velisek L., Sullivan J.A., Upreti C., Kracht J.M., Ericsson M., Wojnarowicz M.W. (2012). Chronic traumatic encephalopathy in blast-exposed military veterans and a blast neurotrauma mouse model. Sci. Transl. Med..

[22] McKee A.C., Stein T.D., Kiernan P.T., Alvarez V.E. (2015). The neuropathology of chronic traumatic encephalopathy. Brain Pathol..

[23] Castillo X., Castro-Obregón S., Gutiérrez-Becker B., Gutiérrez-Ospina G., Karalis N., Khalil A.A., Lopez-Noguerola J.S., Rodríguez L.L., Martínez-Martínez E., Perez-Cruz C. (2019). Re-thinking the Etiological Framework of Neurodegeneration. Front. Neurosci..

[24] Jellinger K.A. (2010). Basic mechanisms of neurodegeneration: A critical update. J. Cell. Mol. Med..

[25] Victoroff J. (2013). Traumatic encephalopathy: Review and provisional research diagnostic criteria. Neuro Rehabil..

[26] Reams N., Eckner J.T., Almeida A.A., Aagesen A.L., Giordani B., Paulson H., Lorincz M.T., Kutcher J.S. (2016). A Clinical Approach to the Diagnosis of Traumatic Encephalopathy Syndrome: A Review. JAMA Neurol..

[27] Montenigro P.H., Bernick C., Cantu R.C. (2015). Clinical features of repetitive traumatic brain injury and chronic traumatic encephalopathy. Brain Pathol..

[28] Hellewell S.C., Beaton C.S., Welton T., Grieve S.M. (2020). Characterizing the Risk of Depression Following Mild Traumatic Brain Injury: A Meta-Analysis of the Literature Comparing Chronic mTBI to Non-mTBI Populations. Front. Neurol..

[29] Stein M.B., Jain S., Giacino J.T., Levin H., Dikmen S., Nelson L.D., Vassar M.J., Okonkwo D.O., Diaz-Arrastia R., Robertson C.S. (2019). Risk of Posttraumatic Stress Disorder and Major Depression in Civilian Patients after Mild Traumatic Brain Injury: A TRACK-TBI Study. JAMA Psychiatr..

[30] McCauley S.R., Boake C., Levin H.S., Contant C.F., Song J.X. (2001). Postconcussional disorder following mild to moderate traumatic brain injury: Anxiety, depression, and social support as risk factors and comorbidities. J. Clin. Exp. Neuropsychol..

[31] Hammond F.M., Corrigan J.D., Ketchum J.M., Malec J.F., Dams-O’Connor K., Hart T., Novack T.A., Bogner J., Dahdah M.N., Whiteneck G.G. (2019). Prevalence of Medical and Psychiatric Comorbidities Following Traumatic Brain Injury. J. Head Trauma Rehabil..

[32] Hai T., Agimi Y., Stout K. (2021). Prevalence of Comorbidities in Active and Reserve Service Members Pre and Post Traumatic Brain Injury, 2017–2019. Mil. Med..

[33] Jennett B., Snoek J., Bond M.R., Brooks N. (1981). Disability after severe head injury: Observations on the use of the Glasgow Outcome Scale. J. Neurol. Neurosurg. Psychiatry.

[34] Ling H., Hardy J., Zetterberg H. (2015). Neurological consequences of traumatic brain injuries in sports. Mol. Cell. Neurosci..

[35] DeKosky S.T., Blennow K., Ikonomovic M.D., Gandy S. (2013). Acute and chronic traumatic encephalopathies: Pathogenesis and biomarkers. Nat. Rev. Neurol..

[36] Cantu R., Sebastianelli W.J., Slobounov S.M. (2006). Concussion Classification: Ongoing Controversy. Foundations of Sport-Related Brain Injuries.

[37] Teasdale G., Jennett B. (1974). Assessment of coma and impaired consciousness. A practical scale. Lancet.

[38] Nakase-Richardson R., Sherer M., Seel R.T., Hart T., Hanks R., Arango-Lasprilla J.C., Yablon S., Sander A., Barnett S., Walker W. (2011). Utility of post-traumatic amnesia in predicting 1-year productivity following traumatic brain injury: Comparison of the Russell and Mississippi PTA classification intervals. J. Neurol. Neurosurg. Psychiatry.

[39] Greenwald B.D., Ambrose A.F., Armstrong G.P. (2012). Mild brain injury. Rehabil. Res. Pract..

[40] Malec J.F., Brown A.W., Leibson C.L., Flaada J.T., Mandrekar J.N., Diehl N.N., Perkins P.K. (2007). The Mayo Classification System for Traumatic Brain Injury Severity. J. Neurotrauma.

[41] Russell W.R., Smith A. (1961). Post-traumatic amnesia in closed head injury. Arch. Neurol..

[42] Ontario Neurotrauma Foundation Guideline For Concussion/Mild Traumatic Brain Injury & Prolonged Symptoms (3rd Edition), For Adults Over 18 Years of Age. https://braininjuryguidelines.org/concussion/.

[43] Reuben A., Sampson P., Harris A.R., Williams H., Yates P. (2014). Postconcussion syndrome (PCS) in the emergency department: Predicting and pre-empting persistent symptoms following a mild traumatic brain injury. Emerg. Med. J..

[44] Jordan B.D. (2013). The clinical spectrum of sport-related traumatic brain injury. Nat. Rev. Neurol..

[45] Omalu B., Bailes J., Hamilton R.L., Kamboh M.I., Hammers J., Case M., Fitzsimmons R. (2011). Emerging histomorphologic phenotypes of chronic traumatic encephalopathy in American athletes. Neurosurgery.

[46] American Psychiatric Association (2013). Diagnostic and Statistical Manual of Mental Disorders: DSM-5.

[47] Martland H.S. (1928). Punch drunk. JAMA.

[48] Castellani R.J., Perry G. (2017). Dementia Pugilistica Revisited. J. Alzheimers Dis..

[49] Heyburn L., Sajja V.S.S.S., Long J.B. (2019). The Role of TDP-43 in Military-Relevant TBI and Chronic Neurodegeneration. Front. Neurol..

[50] Maxwell W.L., Povlishock J.T., Graham D.L. (1997). A mechanistic analysis of nondisruptive axonal injury: A review. J. Neurotrauma.

[51] Medana I., Esiri M. (2003). Axonal damage: A key predictor of outcome in human CNS diseases. Brain.

[52] Zanier E.R., Bertani I., Sammali E., Pischiutta F., Chiaravalloti M.A., Vegliante G., Masone A., Corbelli A., Smith D.H., Menon D.K. (2018). Induction of a transmissible tau pathology by traumatic brain injury. Brain.

[53] Moisse K., Mepham J., Volkening K., Welch I., Hill T., Strong M.J. (2009). Cytosolic TDP-43 expression following axotomy is associated with caspase 3 activation in NFL(−/−) mice: Support for a role for TDP-43 in the physiological response to neuronal injury. Brain Res..

[54] Blaylock R.L., Maroon J. (2011). Immunoexcitotoxicity as a central mechanism in chronic traumatic encephalopathy—A unifying hypothesis. Surg. Neurol. Int..

[55] Klein A.P., Tetzlaff J.E., Bonis J.M., Nelson L.D., Mayer A., Huber D.L., Harezlak J., Mathews V.P., Ulmer J.L., Sinson G.P. (2019). Prevalence of potentially clinically significant MRI findings in athletes with and without sport-related concussion. J. Neurotrauma.

[56] Asken B.M., DeKosky S.T., Clugston J.R., Jaffee M.S., Bauer R.M. (2011). Diffusion tensor imaging (DTI) findings in adult civilian, military, and sport-related mild traumatic brain injury (mTBI): A systematic critical review. Brain Imaging Behav..

[57] Lepage C., Muehlmann M., Tripodis Y., Hufschmidt J., Stamm J., Green K., Wrobel P., Schultz V., Weir I., Alosco M.L. (2019). Limbic system structure volumes and associated neurocognitive functioning in former NFL players. Brain Imaging Behav..

[58] Champagne A.A., Peponoulas E., Terem I., Ross A., Tayebi M., Chen Y., Coverdale N.S., Nielsen P., Wang A., Shim V. (2019). Novel strain analysis informs about injury susceptibility of the corpus callosum to repeated impacts. Brain Commun..

[59] Weis S., Sonnberger M., Dunzinger A., Voglmayr E., Aichholzer M., Kleiser R., Strasser P. (2019). Imaging Brain Diseases A Neuroradiology, Nuclear Medicine, Neurosurgery, Neuropathology and Molecular Biology-Based Approach.

[60] Schultz V., Stern R.A., Tripodis Y., Stamm J., Wrobel P., Lepage C., Weir I., Guenette J.P., Chua A., Alosco M.L. (2018). Age at first exposure to repetitive head impacts is associated with smaller thalamic volumes in former professional american football players. J. Neurotrauma.

[61] Lee J.K., Wu J., Banks S., Bernick C., Massand M.G., Modic M.T., Ruggieri P., Jones S.E. (2017). Prevalence of traumatic findings on routine MRI in a large cohort of professional fighters. AJNR Am. J. Neuroradiol..

[62] Koerte I.K., Mayinger M., Muehlmann M., Kaufmann D., Lin A.P., Steffinger D., Fisch B., Rauchmann B.S., Immler S., Karch S. (2016). Cortical thinning in former professional soccer players. Brain Imaging Behav..

[63] Stone J.R., Avants B.B., Tustison N.J., Wassermann E.M., Gill J., Polejaeva E., Dell K.C., Carr W., Yarnell A.M., LoPresti M.L. (2020). Functional and structural neuroimaging correlates of repetitive low-level blast exposure in career breachers. J. Neurotrauma.

[64] Bazarian J.J., Zhu T., Zhong J., Janigro D., Rozen E., Roberts A., Javien H., Merchant-Borna K., Abar B., Blackman E.G. (2014). Persistent, long-term cerebral white matter changes after sports-related repetitive head impacts. PLoS ONE.

[65] Lee J.K., Wu J., Bullen J., Banks S., Bernick C., Modic M.T., Ruggieri P., Bennett L., Jones S.E. (2020). Association of cavum septum pellucidum and cavum vergae with cognition mood; and brain volumes in professional fighters. JAMA Neurol..

[66] Koerte I.K., Hufschmidt J., Muehlmann M., Tripodis Y., Stamm J.M., Pasternak O., Giwerc M.Y., Coleman M.J., Baugh C.M., Fritts N.G. (2016). Cavum septi pellucidi in symptomatic former professional football players. J. Neurotrauma.

[67] Multani N., Goswami R., Khodadadi M., Ebraheem A., Davis K.D., Tator C.H., Wennberg R., Mikulis D.J., Ezerins L., Tartaglia M.C. (2016). The association between white-matter tract abnormalities, and neuropsychiatric and cognitive symptoms in retired professional football players with multiple concussions. J. Neurol..

[68] Herweh C., Hess K., Meyding-Lamadé U., Bartsch A.J., Stippich C., Jost J., Friedmann-Bette B., Heiland S., Bendszus M., Hähnel S. (2016). Reduced white matter integrity in amateur boxers. Neuroradiology.

[69] Koerte I.K., Ertl-Wagner B., Reiser M., Zafonte R., Shenton M.E. (2012). White matter integrity in the brains of professional soccer players without a symptomatic concussion. JAMA.

[70] Churchill N.W., Caverzasi E., Graham S.J., Hutchison M.G., Schweizer T.A. (2019). White matter during concussion recovery: Comparing diffusion tensor imaging (DTI) and neurite orientation dispersion and density imaging NODDI. Hum. Brain Mapp..

[71] Dettwiler A., Murugavel M., Putukian M., Cubon V., Furtado J., Osherson D. (2014). Persistent differences in patterns of brain activation after sports-related concussion: A longitudinal functional magnetic resonance imaging study. J. Neurotrauma.

[72] Militana A.R., Donahue M.J., Sills A.K., Solomon G.S., Gregory A.J., Strother M.K., Morgan V.L. (2016). Alterations in default-mode network connectivity may be influenced by cerebrovascular changes within 1 week of sports related concussion in college varsity athletes: A pilot study. Brain Imaging Behav..

[73] Han K., Chapman S.B., Krawczyk D.C. (2016). Disrupted intrinsic connectivity among default, dorsal attention, and frontoparietal control networks in individuals with chronic traumatic brain injury. J. Int. Neuropsychol. Soc..

[74] Nordin L.E., Möller M.C., Julin P., Bartfai A., Hashim F., Li T.-Q. (2016). Post mTBI fatigue is associated with abnormal brain functional connectivity. Sci. Rep..

[75] Amyot F., Kenney K., Moore C., Haber M., Turtzo L.C., Shenouda C., Silverman E., Gong Y., Qu B.X., Harburg L. (2018). Imaging of cerebrovascular function in chronic traumatic brain injury. J. Neurotrauma.

[76] Alosco M.L., Tripodis Y., Rowland B., Chua A.S., Liao H., Martin B., Jarnagin J., Chaisson C.E., Pasternak O., Karmacharya S. (2020). A magnetic resonance spectroscopy investigation in symptomatic former NFL players. Brain Imaging Behav..

[77] Lin A.P., Ramadan S., Stern R.A., Box H.C., Nowinski C.J., Ross B.D., Mountford C.E. (2015). Changes in the neurochemistry of athletes with repetitive brain trauma: Preliminary results using localized correlated spectroscopy. Alzheimers Res. Ther..

[78] Beauchamp M.H., Beare R., Ditchfield M., Coleman L., Babl F.E., Kean M., Crossley L., Catroppa C., Yeates K.O., Anderson V. (2013). Susceptibility weighted imaging and its relationship to outcome after pediatric traumatic brain injury. Cortex.

[79] Provenzano F.A., Jordan B., Tikofsky R.S., Saxena C., Van Heertum R.L., Ichise M. (2010). F-18 FDG PET imaging of chronic traumatic brain injury in boxers: A statistical parametric analysis. Nucl. Med. Commun..

[80] Bang S.A., Song Y.S., Moon B.S., Lee B.C., Lee H.Y., Kim J.M., Kim S.E. (2016). Neuropsychological, metabolic, and GABAA receptor studies in subjects with repetitive traumatic brain injury. J. Neurotrauma.

[81] Meabon J.S.R., Huber B.R., Cross D.J., Richards T.L., Minoshima S., Pagulayan K.F., Li G., Meeker K.D., Kraemer B.C., Petrie E.C. (2016). Repetitive blast exposure in mice and combat veterans causes persistent cerebellar dysfunction. Sci. Transl. Med..

[82] Lesman-Segev O.H., La Joie R., Stephens M.L., Sonni I., Tsai R., Bourakova V., Visani A.V., Edwards L., O’Neil J.P., Baker S.L. (2019). Tau PET and multimodal brain imaging in patients at risk for chronic traumatic encephalopathy. NeuroImage Clin..

[83] Mez J., Daneshvar D.H., Kiernan P.T., Abdolmohammadi B., Alvarez V.E., Huber B.R., Alosco M.L., Solomon T.M., Nowinski C.J., McHale L. (2017). Clinicopathological evaluation of chronic traumatic encephalopathy in players of American football. JAMA.

[84] Stein T.D., Montenigro P.H., Alvarez V.E., Xia W., Crary J.F., Tripodis Y., Daneshvar D.H., Mez J., Solomon T., Meng G. (2015). Beta-amyloid deposition in chronic traumatic encephalopathy. Acta Neuropathol..

[85] Zhang H., Schneider T., Wheeler-Kingshott C.A., Alexander D.C. (2012). NODDI: Practical in vivo neurite orientation dispersion and density imaging of the human brain. Neuroimage.

[86] Thompson P.W., Ye L., Morgenstern J.L., Sue L., Beach T.G., Judd D.J., Shipley N.J., Libri V., Lockhart A. (2009). Interaction of the amyloid imaging tracer FDDNP with hallmark Alzheimer’s disease pathologies. J. Neurochem..

[87] Leung K. (2004). 2-(4-(2-[(18)F]Fluoroethyl)piperidin-1-yl)benzo[4;5]imidazo[1,2-a]pyrimidine. Molecular Imaging and Contrast Agent Database (MICAD).

[88] Agdeppa E.D., Kepe V., Liu J., Flores-Torres S., Satyamurthy N., Petric A., Cole G.M., Small G.W., Huang S.C., Barrio J.R. (2001). Binding characteristics of radiofluorinated 6-dialkylamino-2-naphthylethylidene derivatives as positron emission tomography imaging probes for beta-amyloid plaques in Alzheimer’s disease. J. Neurosci..

[89] Harada R., Okamura N., Furumoto S., Tago T., Yanai K., Arai H., Kudo Y. (2016). Characteristics of tau and its ligands in PET Imaging. Biomolecules.

[90] Olivera A., Lejbman N., Jeromin A., French L.M., Kim H.S., Cashion A., Mysliwiec V., Diaz-Arrastia R., Gill J. (2015). Peripheral total tau in military personnel who sustain traumatic brain injuries during deployment. JAMA Neurol..

[91] Shahim P., Tegner Y., Marklund N., Höglund K., Portelius E., Brody D.L., Blennow K., Zetterberg H. (2017). Astroglial activation and altered amyloid metabolism in human repetitive concussion. Neurology.

[92] Shahim P., Tegner Y., Gustafsson B., Gren M., Ärlig J., Olsson M., Lehto N., Engström Å., Höglund K., Portelius E. (2016). Neurochemical aftermath of repetitive mild traumatic brain injury. JAMA Neurol..

[93] Sundman M., Doraiswamy P.M., Morey R.A. (2015). Neuroimaging assessment of early and late neurobiological sequelae of traumatic brain injury: Implications for CTE. Front. Neurosci..

[94] Karantali E., Kazis D., McKenna J., Chatzikonstantinou S., Petridis F., Mavroudis I. (2021). Neurofilament light chain in patients with a concussion or head impacts: A systematic review and meta-analysis. Eur. J. Trauma Emerg. Surg..

[95] Gill J., Mustapic M., Diaz-Arrastia R., Lange R., Gulyani S., Diehl T., Motamedi V., Osier N., Stern R.A., Kapogiannis D. (2018). Higher exosomal tau, amyloid-beta 42, and IL-10 are associated with mild TBIs andchronic symptoms in military personnel. Brain Inj..

[96] Stern R.A., Tripodis Y., Baugh C.M., Fritts N.G., Martin B.M., Chaisson C., Cantu R.C., Joyce J.A., Shah S., Ikezu T. (2016). Preliminary study of plasma exosomal tau as a potential biomarker for chronic traumatic encephalopathy. J. Alzheimers Dis..

[97] Alosco M.L., Tripodis Y., Jarnagin J., Baugh C.M., Martin B., Chaisson C.E., Estochen N., Song L., Cantu R.C., Jeromin A. (2016). Repetitive head impact exposure and later-life plasma total tau in former National Football League players. Alzheimers Dement. Diagn. Assess. Dis. Monit..

[98] Kenney K., Qu B.X., Lai C., CENC Multisite Observational Study Investigators (2018). Higher exosomal phosphorylated tau and total tau among veterans with combat-related repetitive chronic mild traumatic brain injury. Brain Inj..

[99] Alosco M.L., Tripodis Y., Fritts N.G., Heslegrave A., Baugh C.M., Conneely S., Mariani M., Martin B.M., Frank S., Mez J. (2018). Cerebrospinal fluid tau, Aβ, and sTREM2 in former National Football League players: Modelling the relationship between repetitive head impacts, microglial activation, and neurodegeneration. Alzheimers Dement..

[100] Edwards K.A., Greer K., Leete J., Lai C., Devoto C., Qu B.X., Yarnell A.M., Polejaeva E., Dell K.C., LoPresti M.L. (2021). Neuronally-derived tau is increased in experienced breachers and is associated with neurobehavioral symptoms. Sci. Rep..

[101] Singhal A., Baker A.J., Hare G.M.T., Reinders F.X., Schlichter L.C., Moulton R.J. (2002). Association between Cerebrospinal Fluid Interleukin-6 Concentrations and Outcome after Severe Human Traumatic Brain Injury. J. Neurotrauma.

[102] Hensler T., Sauerland S., Riess P., Hess S., Helling H.J., Andermahr J., Bouillon B., Neugebauer E.A. (2000). The effect of additional brain injury on systemic interleukin (IL)-10 and IL-13 levels in trauma patients. Inflamm. Res..

[103] Stein D.M., Lindell A., Murdock K.R., Kufera J.A., Menaker J., Keledjian K., Bochicchio G.V., Aarabi B., Scalea T.M. (2011). Relationship of serum and cerebrospinal fluid biomarkers with intracranial hypertension and cerebral hypoperfusion after severe traumatic brain injury. J. Trauma.

[104] Mohammad N.S., Nazli R., Zafar H., Fatima S. (2022). Effects of lipid based Multiple Micronutrients Supplement on the birth outcome of underweight pre-eclamptic women: A randomized clinical trial. Pak. J. Med. Sci..

[105] Heyser C.J., Masliah E., Samimi A., Campbell I.L., Gold L.H. (1997). Progressive decline in avoidance learning paralleled by inflammatory neurodegeneration in transgenic mice expressing interleukin 6 in the brain. Proc. Natl. Acad. Sci. USA.

[106] Jung Y.J., Tweedie D., Scerba M.T., Greig N.H. (2019). Neuroinflammation as a Factor of Neurodegenerative Disease: Thalidomide Analogs as Treatments. Front. Cell Dev. Biol..

[107] Cheng Y., Pereira M., Raukar N., Reagan J.L., Queseneberry M., Goldberg L., Borgovan T., LaFrance W.C., Dooner M., Deregibus M. (2019). Potential biomarkers to detect traumatic brain injury by the profiling of salivary extracellular vesicles. J. Cell. Physiol..

[108] Oliver J.M., Jones M.T., Kirk K.M., Gable D.A., Repshas J.T., Johnson T.A., Andréasson U., Norgren N., Blennow K., Zetterberg H. (2016). Serum Neurofilament Light in American Football Athletes over the Course of a Season. J. Neurotrauma.

[109] Shahim P., Tegner Y., Wilson D.H., Randall J., Skillbäck T., Pazooki D., Kallberg B., Blennow K., Zetterberg H. (2014). Blood biomarkers for brain injury in concussed professional ice hockey players. JAMA Neurol..

[110] Zetterberg H., Tanriverdi F., Unluhizarci K., Selcuklu A., Kelestimur F., Blennow K. (2009). Sustained release of neuron-specific enolase to serum in amateur boxers. Brain Inj..

[111] Siman R., Toraskar N., Dang A., McNeil E., McGarvey M., Plaum J., Maloney E., Grady M.S. (2009). A panel of neuronenriched proteins as markers for traumatic brain injury in humans. J. Neurotrauma.

[112] Siman R., Shahim P., Tegner Y., Blennow K., Zetterberg H., Smith D.H.D. (2014). Serum SNTF increases in concussed professional ice hockey players and relates to the severity of post-concussion symptoms. J. Neurotrauma.

[113] McMahon P.J., Panczykowski D.M., Yue J.K., Puccio A.M., Inoue T., Sorani M.D., Lingsma H.F., Maas A.I., Valadka A.B., Yuh E.L. (2015). Measurement of the glial fibrillary acidic protein and its breakdown products GFAP-BDP biomarker for the detection of traumatic brain injury compared to computed tomography and magnetic resonance imaging. J. Neurotrauma.

[114] Lewis J.M., Dhawan S., Obirieze A.C., Sarno B., Akers J., Heller M.J., Chen C.C. (2020). Plasma Biomarker for Post-concussive Syndrome: A Pilot Study Using an Alternating Current Electro-Kinetic Platform. Front. Neurol..

[115] McCrea M., Broglio S.P., McAllister T.W., Gill J., Giza C.C., Huber D.L., Harezlak J., Cameron K.L., Houston M.N., McGinty G. (2020). Association of blood biomarkers with acute sport-related concussion in collegiate athletes: Findings From the NCAA and department of defense CARE consortium. JAMA Netw. Open.

[116] Siman R., Giovannone N., Hanten G., Wilde E.A., McCauley S.R., Hunter J.V., Li X., Levin H.S., Smith D.H. (2013). Evidence That the Blood Biomarker SNTF Predicts Brain Imaging Changes and Persistent Cognitive Dysfunction in Mild TBI Patients. Front. Neurol..

[117] Di Battista A.P., Churchill N., Rhind S.G., Richards D., Hutchison M.G. (2019). Evidence of a distinct peripheral inflammatory profile in sport-related concussion. J. Neuroinflamm..

